# Triple negative breast cancer patients presenting with low serum vitamin D levels: a case series

**DOI:** 10.4076/1757-1626-2-8390

**Published:** 2009-07-21

**Authors:** Christa Rainville, Yasir Khan, Glenn Tisman

**Affiliations:** A Medical Corporation13025 Bailey Street, Suite A, Whittier, CA 90601USA

## Abstract

**Introduction:**

Serum vitamin D levels measured as 25-hydroxyvitamin D have been shown to be low in cancer patients, including breast cancer patients. However, the vitamin D status has yet to be studied in different breast cancer phenotypes: luminal A, luminal B, HER2+/ER-, and triple negative comprising the majority of basal-like.

**Case presentation:**

Fifteen triple-negative breast cancer patients have presented to our medical oncology office in the last five years. Thirteen of these fifteen patients (87%) were found to be vitamin D deficient, defined as serum 25(OH)D less than 80 nmol/L, prior to initiation of adjuvant therapy. Ninety-one breast cancer patients from our office were classified as: luminal A (ER+ &/or PR+ and HER2-), luminal B (ER+ &/or PR+ and HER2+), HER2+/ER- (ER-, PR-, and HER2+), and triple-negative or basal-like (ER-, PR-, and HER2-). A normal mean was found from 78 volunteers. The breast cancer patients were found to be statistically different than the normal population. The triple-negative phenotype was found to be the most statistically different than the normal population.

**Conclusion:**

The triple-negative breast cancer phenotype has the lowest average vitamin D level and the highest percentage of patients that are vitamin D deficient. These data suggests that low vitamin D levels are characteristic of the triple-negative phenotype.

## Introduction

Studies have shown that breast cancer incidence, mortality and survival rates are inversely correlated with solar UVB irradiance and/or serum vitamin D levels. In North America, breast cancer mortality rates are highest in the northeast where ultraviolet B radiation levels allow decreased synthesis of vitamin D during a large part of the year. This area tends to have age-adjusted mortality rates that are about 40% higher than in Hawaii and considerably higher than in high sun-exposure regions of the southwest [1].

There are two major forms of vitamin D in the body; 25-hydroxyvitamin D (25(OH)D) and 1,25-dihydroxyvitamin D (1,25(OH)_2_D). 25(OH)D is the storage form of vitamin D. It circulates in the blood and is the best indicator for vitamin D status in the body. 25(OH)D is hydroxylated in the kidney to become 1,25-dihydroxyvitamin D, which is the biologically active form of vitamin D [2].

It was found in 2001 that many tissues are able to directly convert circulating 25(OH)D to 1,25(OH)2D, including mammary tissue [3]. This focuses more attention on the importance of the concentration of the circulating precursor 25(OH)D. It has been shown that 1,25(OH)2D, inhibits cell proliferation and induces apoptosis in breast cancer cells *in vitro*. In animal models, vitamin D analogues slowed down tumor development and promoted regression of established mammary tumors [4]. One group found that mean serum levels of 25(OH)D were significantly lower in breast cancer patients with locally advanced or metastatic disease. They showed that the mean 25(OH)D was statistically lower in advanced-stage breast cancer patients compared to early-stage breast cancer. The authors suggested that the lower serum vitamin D levels might have some causative role in the progression from early-stage to advanced disease as a result of altered gene transcription. They concluded that their findings lend support to the hypothesis that vitamin D has a role in the pathogenesis and progression of breast cancer [5].

Goodwin *et al,* who were measuring all-cause mortality rates in breast cancer patients, found that women who had vitamin D deficiency (<50 nmol/L) when they were diagnosed with breast cancer were 94% more likely to have their cancer metastasize and 73% more likely to die within 10 years. The team also found that only 24% of the women in the study had adequate levels of vitamin D (>72 nmol/L) at the time of the diagnosis [6].

One of the newer phenotypic classifications for breast cancer is based on immunohistochemical and FISH analysis of tumor cell estrogen receptor (ER), progesterone receptor (PR) and HER2 neu (HER2) expression. Luminal A tumors are those that are ER + and/ or PR + and HER2 -. Luminal B tumors are ER + and/or PR + and HER2 +. This definition of luminal B tumors only identifies 30% to 50% that are HER 2 +. The other luminal B tumors with HER2 negativity would be classified under the luminal A subtype. HER2 +/ER- phenotype is HER2+, ER -, and PR -. Approximately 80% of triple negative tumors are basal-like tumors that are ER -, PR -, and HER2 -, a portion of this group is represented by BRCA 1 tumors. We are aware that various investigators have defined similar but not necessarily identical subgroups by including other markers such as tumor grade, cytokeratins and HER1 expression. However, most agree that tumors negative for the three markers HER2, ER and PR (triple negative) belong in the basal-like subgroup [7].

Though low vitamin D levels are associated with advanced stage disease and patients who develop metastases, vitamin D status has yet to be studied within the newly established breast cancer phenotypes. This case series observed low vitamin D levels in thirteen of fifteen patients with the triple negative phenotype.

## Case presentation

Ninety-one newly diagnosed breast cancer patients’ baseline vitamin D levels were obtained from our medical oncology office population in sunny Whittier, California in the last five years. Sixty-five were classified as Luminal A, six were classified as Luminal B, five were classified as HER2+/ER-, and fifteen were classified as triple-negative or basal-like. Baseline vitamin D levels were measured as 25(OH)D prior to any adjuvant therapy. Table 1 shows each patients’ age, ethnicity and baseline vitamin D level as 25(OH)D.

**Table 1.** Triple Negative Patients’ Baseline Vitamin D Level, Age and Ethnicity

Classification	Baseline Serum 25(OH)D (nmol/L)	Age	Ethnicity
Luminal A	125.0	51	WNH
Luminal A	49.0	81	WNH
Luminal A	107.0	67	WNH
Luminal A	63.0	83	WNH
Luminal A	33.7	75	WNH
Luminal A	102.0	83	WH
Luminal A	98.0	65	WNH
Luminal A	59.3	53	WNH
Luminal A	28.0	61	WNH
Luminal A	123.9	76	WNH
Luminal A	64.0	63	WNH
Luminal A	54.0	66	WNH
Luminal A	112.2	85	WNH
Luminal A	92.0	62	WNH
Luminal A	60.0	69	WNH
Luminal A	31.2	90	WNH
Luminal A	96.0	74	A
Luminal A	95.4	72	WHN
Luminal A	300.0	75	WHN
Luminal A	74.7	50	WHN
Luminal A	50.0	69	WHN
Luminal A	53.0	65	WHN
Luminal A	11.7	80	WHN
Luminal A	101.0	64	WHN
Luminal A	84.0	80	WHN
Luminal A	66.6	74	WHN
Luminal A	86.0	62	WHN
Luminal A	125.0	39	WH
Luminal A	117.0	69	WNH
Luminal A	61.0	80	B
Luminal A	36.0	74	WNH
Luminal A	16.0	84	WNH
Luminal A	61.0	59	WNH
Luminal A	82.0	68	WNH
Luminal A	9.0	107	WNH
Luminal A	57.6	87	WNH
Luminal A	72.0	79	WNH
Luminal A	213.6	77	WNH
Luminal A	35.0	78	WNH
Luminal A	68.0	49	B
Luminal A	50.0	63	WNH
Luminal A	67.0	84	WNH
Luminal A	44.0	83	WNH
Luminal A	208.8	79	WNH
Luminal A	81.0	90	WNH
Luminal A	105.0	67	WNH
Luminal A	23.5	72	WNH
Luminal A	56.4	70	WNH
Luminal A	23.0	61	WNH
Luminal A	34.3	55	WH
Luminal A	65.0	80	WNH
Luminal A	88.0	81	WNH
Luminal A	14.0	73	WNH
Luminal A	91.0	52	WH
Luminal A	68.0	55	WNH
Luminal A	99.0	68	WNH
Luminal A	61.0	77	WNH
Luminal A	185.1	62	WH
Luminal A	18.4	63	WNH
Luminal A	65.5	48	WNH
Luminal A	75.1	83	WNH
Luminal A	89.0		WNH
Luminal A	238.1	67	WNH
Luminal A	39.0	87	WNH
Luminal A	97.5	66	WNH
Luminal B	82.2	59	WNH
Luminal B	76.9	60	B
Luminal B	48.6	81	WHN
Luminal B	41.0	35	WHN
Luminal B	84.0	66	A
Luminal B	117.0	68	WNH
HER2/neu +	74.0	63	WNH
HER2/neu +	94.0	81	WNH
HER2/neu +	60.8	60	WNH
HER2/neu +	114.4	52	WNH
HER2/neu +	121.1	54	WNH
Triple-Negative	49.6	52	WNH
Triple-Negative	61.0	66	WH
Triple-Negative	38.0	34	WNH
Triple-Negative	22.7	89	WNH
Triple-Negative	34.7	83	WNH
Triple-Negative	45.0	79	WNH
Triple-Negative	55.0	58	WNH
Triple-Negative	77.2	76	WH
Triple-Negative	25.0	43	WNH
Triple-Negative	82.4	45	WNH
Triple-Negative	30.0	71	WNH
Triple-Negative	72.0	69	WNH
Triple-Negative	41.6	57	WNH
Triple-Negative	80.0	79	WNH
Triple-Negative	32.4	55	WNH

A, Asian; B, Black; WH, White Hispanic; WNH, White Not-Hispanic.

Blood was obtained for vitamin D assay from non-fasting patients in standard serum separator vacutainer tubes with gel and clot activator and allowed to clot at room temperature (Franklin Lakes, NJ) [8]. After 15-30 minutes the tubes were centrifuged and serum was separated and stored frozen at -40 degrees centigrade for less than 2 weeks.

An Immunodiagnostic System (IDS) 25-hydroxyvitamin D kit by EIA method was used on a DSX system analyzer by DYNEX. We are currently involved in a program by Vitamin D External Quality Assessment Scheme (DEQAS) based in London that assesses the accuracy of our 25(OH)D levels compared to laboratories across the world. Our laboratory reached to performance target set by the DEQAS Advisory Panel for the 2007-2008 year.

Thirteen out of fifteen (87%) of the triple negative patients were vitamin D deficient as defined as less than 80 nmol/L [9]. The other two patients were very close to being deficient, having vitamin D levels of 80.0 nmol/L and 82.4 nmol/L. When looking at all of our breast cancer patients (91 total), we found that 54 patients (62%) had baseline vitamin D levels in the deficiency range <80 nmol/L. Of our 91 breast cancer patients, 65 were found to belong to the Luminal A subtype (ER + and/ or PR + and HER2 -), six were Luminal B subtype (ER + and/or PR + and HER2 +), five were the HER2+/ER- subtype (HER2+, ER -, and PR -), and 15 were classified as the triple-negative subtype (ER-, PR-, HER2-). A normal control population was established from a community outreach program to Whittier, CA non-hospitalized residents, which included 78 volunteers that did not have cancer. Table 2 shows the mean, median, standard deviation and percent deficient for each group. The mean ± standard deviation for serum vitamin D levels were as follows: normal volunteers (90 ± 40 nmol/L), breast cancer patients (76 ±50), luminal A (79 ± 50 nmol/L), luminal B (75 ± nmol/L 30), HER2+/ER- (93 ± 30 nmol/L), and triple-negative or basal-like (50 ± 20 nmol/L). The triple negative phenotype had the lowest average and median baseline vitamin D level and had the highest percentage with vitamin D deficiency.

**Table 2.** Sample Size, Mean 25(OH)D, Median 25(OH)D, Standard Deviation, % Vitamin D Deficient of Normal Controls and Breast Cancer Classifications

	Normal Control	Breast Cancer	Luminal A	Luminal B	Her2+/ER-	Triple-Negative
Sample Size	78	91	65	6	5	15
Mean Baseline Serum 25(OH)D (nmol/L)	90	76	79	75	93	50
Median Baseline Serum 25(OH)D (nmol/L)	86.0	66.6	67.0	79.5	94.0	45.0
Standard Deviation Baseline Serum 25(OH)D (nmol/L)	40	50	50	30	30	20
% Vitamin-D Deficient	43%	62%	58%	50%	40%	87%

To assess whether vitamin D levels were statistically lower in cancer patients than normal and whether vitamin D differed by tumor stage we used the unpaired t-test with significance level, α, of 0.05 (Table 3). The unpaired t-test showed that breast cancer patients have significantly lower vitamin-D levels than normal, p < 0.015 and we could not find statistical difference by tumor stage. Further analysis of all breast cancer groups with normal volunteers by one-way ANOVA identified statistically different groups (F = 2.56 & Significance value = 0.041). Data is shown in Table 4. Furthermore, we used ANOVA to identify the most statistically different breast-cancer type. Post-hoc analysis by Tukey Honestly Statistically Different (HSD) test, Table 5, was used to identify which type of breast cancer was the most statistically different than normal. The triple-negative subgroup was found to be the most statistically different than the normal volunteers (significance value = 0.01).

**Table 3.** Un-Paired T-test

	Normal vs. Breast Cancer	Early vs. Advanced
Degrees of Freedom	164	85
t-critical	1.65	-1.66
Significance Value (p)	0.011	0.101

**Table 4.** Anova- Single Factor Comparing Difference Between Groups

Anova	F-critical	Significance Value (p)
Between Groups	2.43	0.02

**Table 5.** Tukey HSD Comparing Normal Control to Breast Cancer

Tumor Classifications	Mean Difference	Significance Value (p)
Normal Control vs. Luminal A	10.59	0.588
Normal Control vs. Luminal B	15.06	0.923
Normal Control vs. Her2+/ER-	-2.86	1
Normal Control vs. Triple-Negative	40.23	0.01

## Conclusion

The finding that breast cancer patients with the lowest serum 25(OH)D levels presented with the biologically aggressive triple-negative tumor phenotype was not a surprise. Others have demonstrated by *in vitro* studies that breast tumor cell lines growing in the presence of limited vitamin D frequently over-express more aggressive phenotypes associated with poor prognosis [10]. Compared with luminal A, triple-negative (basal-like) tumors had more TP53 mutations (44% vs 15%), higher mitotic index, more marked nuclear pleomorphism and higher combined grade as well as poor cancer-specific survival [7].

This case series found that patients with the more aggressive triple-negative phenotype had a mean serum vitamin D level of 50 nmol/L compared to a mean of 90 nmol/L for normal Whittier, CA volunteers. The assay normal cut-off was defined as >=80 nmol/L [9]. This finding, coupled with tissue culture experiments and the epidemiological study noted previously, suggests that the serum vitamin D level may be important in tumor development and phenotypic expression and the biologic behavior of breast tumors. This hypothesis is compatible with the fact that African American women have the highest breast cancer specific mortality rates, the lowest serum levels of 25(OH)D, and the highest incidence of aggressive triple-negative or basal-like tumors (39%) [7].

This series observed that triple-negative breast cancer patients have lower vitamin D levels than the other breast cancer phenotypes. In addition, we found that the triple-negative subtype is the most statistically different than normal compared to the other subtypes. The lack of vitamin D transport into cells may contribute to the phenotypic expression. Further studies are warranted to investigate possible relationships between the breast cancer phenotypes, pathological grades, clinical stages, and overall and cancer specific survival and vitamin D sufficiency. We think it prudent to supplement all patients with breast cancer and low levels of vitamin D with adequate amounts of vitamin D_3_ and generally administer 2000 IU/day orally. This dose in combination with moderate sunlight is usually enough to raise serum 25(OH)D levels to 130 nmol/L, which is associated with a 50% reduction in incidence of breast cancer, according to observational studies [11].

## Abbreviations

ER: estrogen receptor
HER2: her2/*neu* receptor
PR: progesterone receptor
1,25(OH)_2_D: 1,25-dihydroxyvitamin D
25(OH)D: 25-hydroxyvitamin D
